# Pneumatic unidirectional cell stretching device for mechanobiological studies of cardiomyocytes

**DOI:** 10.1007/s10237-019-01211-8

**Published:** 2019-08-23

**Authors:** Joose Kreutzer, Marlitt Viehrig, Risto-Pekka Pölönen, Feihu Zhao, Marisa Ojala, Katriina Aalto-Setälä, Pasi Kallio

**Affiliations:** 1 Micro-and Nanosystems Research Group, Faculty of Medicine and Health Technology, Tampere University, Korkeakoulunkatu 3, 33720 Tampere, Finland; 2 Department of Health Technology, Technical University of Denmark, 2800 Lyngby, Denmark; 3 Heart Group, Faculty of Medicine and Health Technology, Tampere University, Arvo Ylpön Katu 34, 33520 Tampere, Finland; 4 Department of Biomedical Engineering, Eindhoven University of Technology, 5600 MB Eindhoven, The Netherlands; 5 Heart Centre, Tampere University Hospital, Arvo Ylpön Katu 6, 33520 Tampere, Finland

**Keywords:** Mechanical stimulation, Cardiomyocytes, hiPSC, PDMS

## Abstract

In this paper, we present a transparent mechanical stimulation device capable of uniaxial stimulation, which is compatible with standard bioanalytical methods used in cellular mechanobiology. We validate the functionality of the uniaxial stimulation system using human-induced pluripotent stem cells-derived cardiomyocytes (hiPSC-CMs). The pneumatically controlled device is fabricated from polydimethylsiloxane (PDMS) and provides uniaxial strain and superior optical performance compatible with standard inverted microscopy techniques used for bioanalytics (e.g., fluorescence microscopy and calcium imaging). Therefore, it allows for a continuous investigation of the cell state during stretching experiments. The paper introduces design and fabrication of the device, characterizes the mechanical performance of the device and demonstrates the compatibility with standard bioanalytical analysis tools. Imaging modalities, such as high-resolution live cell phase contrast imaging and video recordings, fluorescent imaging and calcium imaging are possible to perform in the device. Utilizing the different imaging modalities and proposed stretching device, we demonstrate the capability of the device for extensive further studies of hiPSC-CMs. We also demonstrate that sarcomere structures of hiPSC-CMs organize and orient perpendicular to uniaxial strain axis and thus express more maturated nature of cardiomyocytes.

**Electronic supplementary material:**

The online version of this article (10.1007/s10237-019-01211-8) contains supplementary material, which is available to authorized users.

## Introduction

Human-induced pluripotent stem cell-derived cardiomyocytes (hiPSC-CMs) have large potential in regenerative biomedicine particularly for disease modeling, drug screening, personalized medicine and cell therapies (Denning et al. 2016; Bedada et al. 2016; Sun and Nunes 2017). However, hiPSC-CMs in vitro differ from the phenotype of adult cardiomyocytes (CMs). Fully mature hiPSC-CMs have not been obtained to date, and they are considered structurally and functionally immature. A substantial effort has been placed on the use of biochemical, biophysical, genetic, and environmental inducers to improve structural and functional properties of hiPSC-CMs toward those seen in adult CMs (Denning et al. 2016; Sun and Nunes 2017). Typically, specific biochemical inducers (culture medium with supplements) are applied alone to control the maturity, which seems to be an insufficient method. Therefore, other cues, such as biophysical cues, are needed to further improve hiPSC-CMs maturation.

Biophysical cues, such as mechanical cues, play an important role when mimicking cellular microenvironment in vitro (Bukoreshtliev et al. 2013). Mechanobiological studies are essential to further understand the sensing and responding mechanisms of cells to mechanical signals. The effect of different mechanical stimuli on different cell types has been extensively studied and shown to affect, for example, cell morphology, orientation, focal adhesion, chemical signaling pathways and the fate of differentiated stem cells in a dish. To demonstrate the effect of mechanical stimuli, a variety of different engineering approaches, such as flow-induced shear forces, hydrostatic pressure, cell indentation, substrate topography, substrate stiffness, and substrate stretching, have been widely reported and reviewed (Gwak et al. 2008; Kim et al. 2009; Ghafar-zadeh et al. 2011; Maul et al. 2011; Lee et al. 2011; Simmons et al. 2012; Kamble et al. 2016).

Substrate stretching has been one of the biophysical cues applied when studying the mechanobiology of hiPSCs. There are numerous studies reported that concentrate specifically on substrate stretching (Naruse et al. 1998; Jungbauer et al. 2008; Huh et al. 2010; Huang et al. 2010; Wan et al. 2011; Huang and Nguyen 2013; Shao et al. 2013; Kreutzer et al. 2014; Mihic et al. 2014; Tremblay et al. 2014; Wang et al. 2016; Kamble et al. 2018). Also, few commercial platforms are available on the market specialized on substrate stretching (Flexcell^®^, Strex Inc, CellScale). A unidirectional stretchable substrate in particular enables the mechanical stimulation of cells with biomimetic contraction and relaxation phases. These phases can further “train” cells to reach more maturated state, e.g., structurally elongated and thus more matured cardiomyocytes in a dish. Furthermore, unidirectional stretching platforms have been widely reported to align cells (Naruse et al. 1998; Lee et al. 2007; Jungbauer et al. 2008; Shao et al. 2013; Wang et al. 2016; Kamble et al. 2018). Therefore, unidirectional stretching platforms are good research tools to achieve a more biomimetic nature of cardiomyocytes, for example. The aligned and elongated cells express more organized sarcomere structures that present one of the many characteristics of adult CMs (Denning et al. 2016). In addition to the more organized sarcomere structures, CMs’ morphology changes from polygonal to rod-shaped, calcium cycling refines, and their spontaneous beating is turned off when the maturation develops (Denning et al. 2016; Bedada et al. 2016). Therefore, it is essential to visualize cells and their response to mechanical stimulation with various high-resolution real-time imaging modalities during the stretching and possible combine different imaging modalities (Ahola et al. 2018) to achieve better understanding of cells

In general, several approaches have been applied to implement unidirectional stretching. Two major approaches to create the actuation force to stretch the membrane underneath the cells include electromechanical and pneumatic actuation. A majority of the developed systems have used an electric actuator, such as a stepper motor, a DC motor, or a voice coil actuator to deform the cell cultivation membrane (Naruse et al. 1998; Pfister et al. 2003; Jungbauer et al. 2008; McMahon et al. 2008; Huang et al. 2010; Kluge et al. 2011; Wan et al. 2011; Shao et al. 2013). Also, companies such as Strex Inc. and CellScale are utilizing motor-controlled actuation. The electromechanical actuation systems are typically bulky and complex, especially when operating a number of individual parallel wells. In general, electromechanical actuators and electronics can also affect the sensitive environment inside an incubator and thus giving rise to temperature or increase a contamination risk.

Only few groups have reported the use of pneumatic systems for unidirectional cell stretching, in which a partial vacuum pressure is used to stretch the cell culture substrate (Wang et al. 2010; Huh et al. 2010; Huang and Nguyen 2013; Kreutzer et al. 2014; Tremblay et al. 2014; Kamble et al. 2018). Furthermore, the commercial product from Flexcell^®^ relies on partial vacuum actuation. A major advantage to apply partial vacuum pressure is the use of nontoxic and sterilizable materials that enable cultures placed inside the incubator. Also high-throughput systems are easier to implement using the pneumatic approach (Moraes et al. 2010; Kamble et al. 2018, Flexcell^®^).

Typical pneumatically actuated stretching devices require lubricants, such as grease or oil, at the bottom of the membrane (Wang et al. 2010, Flexcell^®^). Some lubricants are claimed to react or penetrate through a permeable silicone-based stretching membranes, which can affect the cells atop (Huang and Nguyen 2013). Lubricants also disturb live cell imaging with inverted microscopes through the stretchable membrane. For example, a commercial stretching system, Flexcell^®^, requires lubricants to slide the membrane on top of a loading post. That disturbs imaging due to the remains of lubricants when the membrane is replaced from the stretching platform for optical microscopy studies. Lubricants were also used in the work by Wang et al. (2010), who introduced a pneumatic equi- and uniaxial stretching system capable of high resolution imaging during the stretching. Furthermore, lubricants could make the usability of the system difficult and multiple interfaces between optics and the cell layer affect the imaging quality. Huh et al. (2010) introduced a unidirectional stretching system that enables culturing cells on both sides of a stretchable membrane. That requires, however, continuous or frequent medium change as the cells are cultured in a small perfusion channel. The system also includes multiple interfaces that affect the imaging quality. A similar system was introduced by Tremblay et al. (2014), who were able to produce both equi- and uniaxial stretching but in a small reaction chamber that also requires continuous or frequent medium change.

In this paper, we introduce a vacuum-operated uniaxial stretching device that enables high-resolution live cell phase contrast and fluorescent microscopy during stretching. The cell stretching device includes a large medium container (600 µl) to provide a long-term nutrient supply. Multiple parallel (up to 24 used) devices can be simultaneously stretched inside an incubator. The stretching device does not include corrosive parts or a loading post and does not require any lubrication but provides purely planar membrane deformation to study cells under a uniaxial strain. In the paper, we describe the implementation of the stretching device made of polydimethylsiloxane (PDMS) elastomer and characterize its functionality utilizing computational simulations and experimental validation. Computational simulations also show that the fluid movements inside the stretching device do not create any other mechanical cues, and therefore, mechanical substrate stretching is the only mechanobiological stimulation valid for the cells. Furthermore, we demonstrate for the first time the combination of human cardiomyocyte stretching with video-based beating analysis and calcium imaging through a stretchable membrane. This demonstration reveals that the proposed unidirectional stretching device provides favorable imaging properties to study live cells in details using different imaging modalities.

## Materials and methods

### Implementation and operation of the uniaxial stretching device

**Fig. 1:**
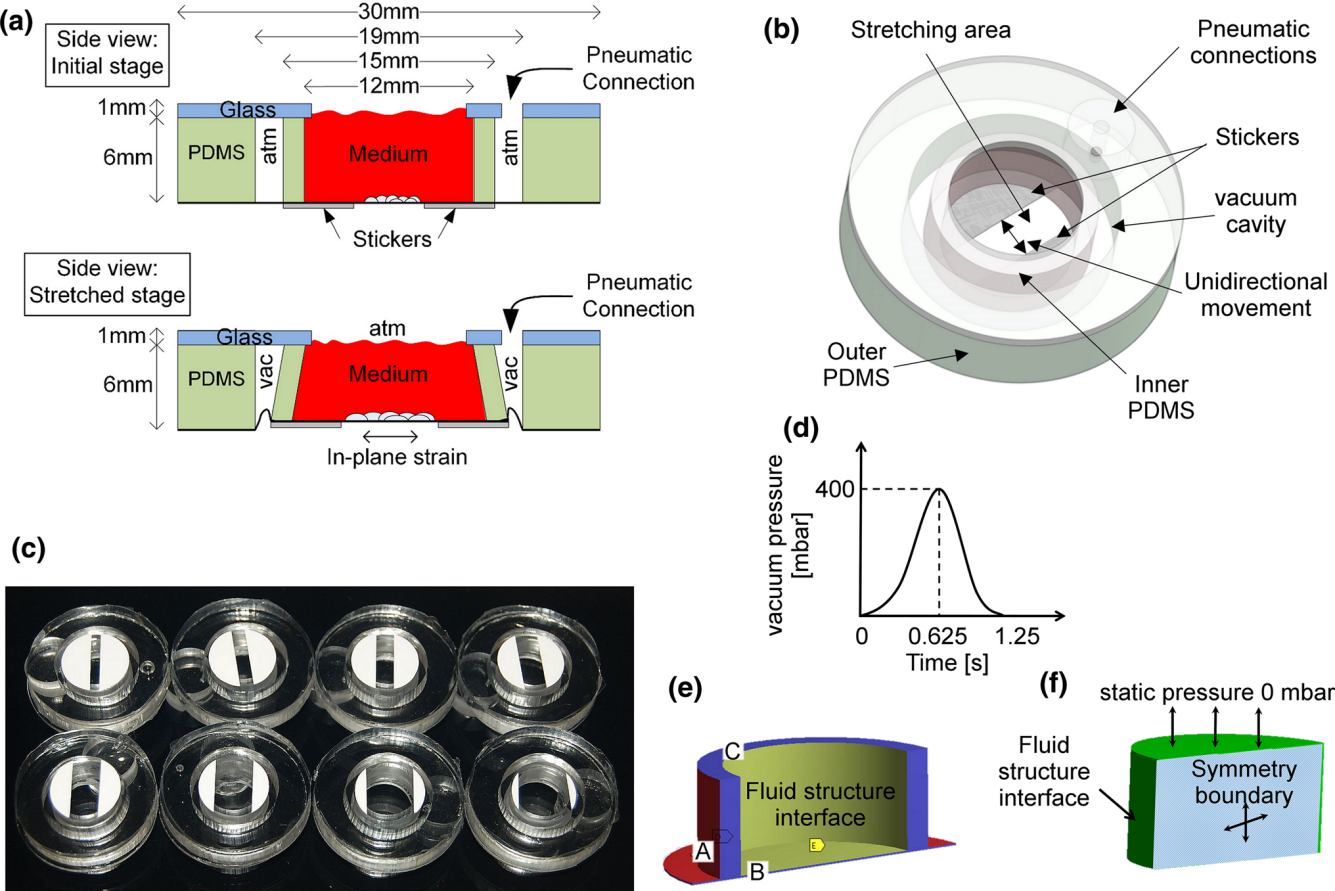
**a** Side view of a stretching device in its initial state and stretched stage. **b** An example of a stretching device with 5-mm gap between the restricted areas. **c** Different stretching devices (membrane side up) with stickers attached on the bottom of the membrane. The gap between stickers defines the stretching area. Gap sizes 2 mm (top left), 3 mm, 4 mm, 5 mm, 6 mm (bottom left), 8 mm, 10 mm and 12 mm. **d** Pulsatile pressure loading profile (0.8 Hz; 400 mbar) was used for simulations and experiments. Boundary conditions of the fluid–structure interaction model were defined by **e** solid domain and **f** fluid domain

The design of the uniaxial stretching device is modified from the principle of an equiaxial stretching device developed earlier by Kreutzer et al. (2014). Briefly, a partial vacuum pressure is used for controlling the stretching device by applying vacuum pressure in a closed cavity between two polydimethylsiloxane (PDMS) shells, as shown in Fig. 1a, b. The vacuum pressure deforms and expands the inner shell. This further deforms the membrane attached to the bottom of the shell and stretches the cells cultivated on the membrane. To change the strain mode from the previously reported equiaxial strain to the uniaxial strain, two rigid structures are attached underneath the PDMS membrane. The rigid structures are fabricated from polyvinyl chloride (PVC) foil stickers (Megaprint Ky, Helsinki, Finland). Rigid stickers having a shape of circular segments cover partially the cell cultivation area and the inner PDMS shell, as shown in Fig. 1a–c.

In the unidirectional mode, the rigid circular segment structures guide the deformation of the PDMS membrane. The PDMS membrane on top of the vinyl foils does not stretch but moves alongside the inner shell when the shell deforms. This results in a unidirectional strain between the rigid structures. In this study, the gap between the two rigid structures, called a stretching area, varies from 2 to 12 mm (See Fig. 1c). The gap width affects the strain of the membrane and wall shear stress (WSS), which were characterized for all structures utilizing computational simulations. The simulated strain of the membrane was experimentally validated using a particle tracking method.

The uniaxial stretching devices were prepared utilizing PDMS (Sylgard 184, Dow Corning, USA), glass plates and vinyl foil stickers. The preparation of the PDMS parts has been described in detail previously by Kreutzer et al. (2014). Briefly, silicone elastomer pre-polymer (base) and cross-linker (curing agent) were combined in a 10:1 (w/w %) mixing ratio. The mixture was poured in a mold, degassed, and cured in an oven for 10 h at 60 °C. The shell structures were punched from 6-mm-thick bulk elastomer using custom-made tools. The PDMS shells, PDMS membranes, and glass plates were assembled using oxygen plasma (Pico-SR-PCCE, Diener Electronic GmbH, Ebhausen, Germany) using the following parameters: pressure of 0.3 mbar, power of 30 W, and duration of 20 s for PDMS–PDMS bonding and 15 s for PDMS–glass bonding. The vinyl foils were attached underneath the membrane with the help of oxygen plasma using the same parameters as in PDMS–PDMS bonding.

### Vacuum operation system

The partial vacuum pressure was generated using an in-house developed system described in detail by Kreutzer et al. (2014). The partial vacuum pressure was computer controlled with Labview-based software that allows a variety of applied strain forms and operation sequences. In cell experiments, cyclic sinusoidal strain forms were applied, while in experiments to characterize the membrane strain static partial pressures (0–400 mbar) were used.

### Computational simulation

To quantify the mechanical strain field and wall shear stress on the membrane, a two-way fluid–structure interaction (FSI) model of the cell stretching device was developed using ANSYS Multiphysics platform (ANSYS, Inc., US). Specifically, we investigated the deformation of the structure (especially the substrate membrane) and the behavior of medium flow on the membrane under dynamic loading, i.e., sinusoidal loading (*f* = 0.8 Hz; *A* = 400 mbar peak to peak) as illustrated in Fig. 1d. We utilized axial-symmetry and modeled a half of the structure in this study. As the membrane and the cylindrical shells were fabricated from PDMS, we used Young’s modulus of 2 MPa and Poisson’s ratio of 0.49 (Zhao et al. 2014). The rigid structures underneath the substrate were made of PVC, and thus, we applied Young’s Modulus of 3.3 GPa and Poisson’s ratio of 0.4. To mimic the vacuum pressure, we applied a normally outward pressure on the wall of the inner PDMS shell and the surface of the membrane, shown as Surface A (red color) in Fig. 1e. Since the top of the inner PDMS shell was attached to the glass substrate, Surface C (Fig. 1e) was set to be fixed in the simulation model. Similarly, the PDMS membrane was attached to the thick outer PDMS shell, and thus, the edge of the membrane was assumed to be fixed in the model. The symmetric Surface B was defined as frictionless support. In addition, the contact between the rigid PVC layers and the PDMS membrane was defined as bonded, which followed a pure penalty formulation. Moreover, inside the culture well, the surfaces that interacted with the culture medium were defined as the fluid–structure interface (yellow areas in Fig. 1e). The entire solid domain was meshed by a tetrahedron method with a patch conforming algorithm. We applied a uniform element size (200 µm) to the entire solid domain and simulated the system in a transient state by a finite element (FE) approach.

In the fluid domain, the culture medium in the computational fluid dynamics (CFD) domain was assumed to possess the properties of water, having a dynamic viscosity of 1 mPa s and a density of 1000 kg/m^3^ (37 °C). A laminar flow type was applied for defining the flow. As shown in Fig. 1f, the top surface was defined as the open boundary with a static pressure of 0 mbar. The bottom and side surfaces formed the fluid–structure interfaces between the CFD and FE domains, and the symmetric surface (in shadow in Fig. 1f) was defined as the symmetry boundary. This two-way FSI analysis followed a staggered iteration approach. In this approach, the fluid equations were solved and the resulting fluid stress tensor acting at the fluid–solid interfaces was applied as the boundary condition on the solid domain. The resulting deformation in the solid domain was relayed back to the fluid domain and the solution continued through further iterations until a convergence criteria was reached. The fluid domain was meshed by a tetrahedron method with a patch conforming algorithm. Moreover, the mesh with an element size of 200 µm was applied on the fluid domain, while the fluid–structure interfaces were refined with an element size of 80 µm. Since the geometry of the fluid domain in different structures was the same, approximately 354,530 elements discretized the fluid domain. Finally, the model was resolved in a transient state (time step 0.0125 s) by ANSYS CFX solver under the RMS residual convergence criteria of 1.0 × 10^−4^.

### Experimental characterization of the strain field

The in-plane strain field of the deforming membrane was characterized utilizing displacement calculation algorithms. Green fluorescent polystyrene (PS) nanoparticles (*d* = 4.18 µm ± 0.397 µm, *c* = 6 µl/ml in DI water, Dragon Green, Bangs Laboratories Inc.) were unspecifically absorbed to the PDMS membrane and were used as landmarks to follow the deformation. The membrane was stretched using nine static partial vacuum pressure settings ranging from 0 to 400 mbar in 50 mbar increments. The membrane deformation at each applied partial vacuum pressure was imaged with an inverted optical microscope system (Nikon TS100, Nikon Inc.) using 4x magnification and 5Mpix CCD camera (AVT Manta G-504, Allied Vision Technologies GmbH, Stadtroda, Germany).

In-plane strain components for *x*-direction and *y*-direction were calculated utilizing particle tracking algorithms (Sbalzarini and Koumoutsakos 2005) of Fiji (Schindelin et al. 2012) (open source image processing software) to track the coordinates of the particles. Ten particles were selected from the region of interest on the membrane and *x*- and *y*-strain components were calculated between each particle pair for each pressure. Particle pairs where particles located too close (< 300 pixels) to each other were discarded from the calculations because of a probability for a pixelation error.

### Surface functionalization

Surface functionalization is needed to render the initially hydrophobic surface of PDMS to be suitable for the attachment of extracellular matrix (ECM) proteins and eventually cells. In these experiments, we used a modified version of the covalent functionalization protocol recently introduced by Leivo et al. (2017). Briefly, a three-step covalent functionalization procedure was applied targeting the nitrogen-rich molecular sidechains of extracellular matrix proteins (such as gelatin) to bind permanently onto the substrate. The procedure ensures a full-coverage and functionality of the protein coating on the substrate and ultimately provides durable and long-lasting cellular attachment during the mechanical stretching. First, in order to create hydroxyl groups to the PDMS surface, the cultivation area of the completely assembled stretching device was activated by using oxygen plasma (PDMS-PDMS setting, see Section “Implementation and operation of the uniaxial stretching device”). Immediately after the plasma treatment, 10% (3-aminopropyl) triethoxysilane (APTES, Sigma-Aldrich GmbH, Germany) in methanol was applied to the cultivation chamber and incubated for 2 min. After a double rinse with DI water, the device was left to dry completely. Second, 1 mg/ml l-Ascorbic Acid (l-AA, Sigma-Aldrich GmbH, Germany) in 1xPBS was used as a spacer reagent and incubated for 1 h. After incubation, the device was rinsed three times with PBS. Third, 0.1% gelatin (Sigma-Aldrich GmbH, Germany) in 1xPBS was applied and incubated for 1 h. After three times of applying the washing procedure with PBS, the device was ready for the experimental use.

### Patient-specific human iPSC lines and cardiomyocyte differentiation

The collection of biopsies for generating patient-specific human-induced pluripotent stem cell (hiPSC) lines was approved by the ethical committee of Pirkanmaa Hospital District (Aalto-Setälä R08070), and written informed consent was obtained from all the donors. Human iPSC lines were established by sendai viral (CytoTune^®^ iPS reprogramming kit, Thermo Fisher Scientific, Waltham, MA, USA) or retroviral transfection of *OCT3/4*, *SOX2*, *KLF4* and *c*-*MYC* (Ohnuki et al. 2009). The characterization of hiPSC lines was done as described by Lahti et al. (2012).

Human iPSCs were cultured on mitomycin-C inactivated mouse embryonic fibroblasts (26,000 cells/cm^2^, CellSystems Biotechnologie Vertrieb GmbH, Troisdorf, Germany). Fibroblasts act as feeder cells to support the growth of undifferentiated hiPSCs and to maintain the pluripotent state of hiPSCs in maintenance medium (KO-DMEM (Thermo Fisher Scientific) base, 20% KO-SR (Thermo Fisher Scientific), 1% nonessential amino acids (NEAA, Lonza Group Ltd, Basel, Switzerland), 2 mM GlutaMax (Thermo Fisher Scientific), 50 U/ml penicillin/streptomycin (Lonza), 0.1 mM 2-mercaptoethanol (Thermo Fisher Scientific) and 4 ng/ml basic fibroblast growth factor (bFGF, Peprotech, Rocky Hill, NJ, US)). The medium was changed three times per week.

Differentiation into cardiomyocytes was performed by either co-culturing hiPSCs with murine visceral endoderm-like (END-2) cells (Mummery et al. 2003) or via small molecule modulation of canonical Wnt signaling (Lian et al. 2012). After 15 days, hiPSCs formed spontaneously beating clusters which were cut and dissociated with collagenase A (Roche Diagnostics, Basel, Switzerland) (Mummery et al. 2003).

### Experimental culture platforms and mechanical loading of stretching devices

In cell experiments, hiPSC-derived cardiomyocytes were seeded onto three different test structures: active stretching devices (5 mm gap width used for all cell experiments) and non-stretched PDMS wells and 4-well polystyrene cell culture plates (Nunc) for controls. All of the structures were coated with 0.1% gelatin (Sigma). Dissociated (Collagenase A, Sigma) cardiomyocytes were plated on the structures and let to attach at 37 °C for 45 min before loading up to the final amount of EB medium (500 µl/PDMS device and 1 ml per well in 4-well plate): KO-DMEM base, 20% FBS (Thermo Fisher Scientific), 1% nonessential amino acids, 2 mM GlutaMax and 50 U/ml penicillin/streptomycin. Cells were cultured in the incubator for 3 days before starting the stretching (Day 3). The stretching experiments were performed inside an incubator. EB medium was changed on Day 3 and Day 7.

A pre-stretching protocol was used to adapt the cells for the stretching and to avoid detaching the cells due to the sudden maximal uniaxial strain on substrate. The protocol was adapted from our previous work (Kreutzer et al. 2014) where we demonstrated that the pre-stretching protocol is useful to have at the beginning to avoid detaching the cells. The pre-stretching protocol was applied at the beginning of the stretching with the following parameters: *f* = 0.5 Hz; 1.5% for 1 min, 3% for 2 min and 8% for 7 min. After pre-stretching, cells were stretched with sinusoidal cyclic stress of 8% elongation at a frequency of 0.8 Hz for 2, 4 or 7 days. Hereby, each experimental round lasted 10 days including nine individual stretching devices stretched inside the incubator. Three stretching devices and three non-stretched PDMS controls were analyzed in each of the following time points Day 5, Day 7 and Day 10 (corresponding day 2, 4 and 7 of stretching) and, in addition, three non-stretched PDMS controls were analyzed before the stretching (Day 3). To validate the correct cell behavior through the experiments and to obtain additional information, the cells on 4-well plate controls were analyzed only in the last time points. After finishing the stretching, samples were fixed immediately with 4% PFA (Sigma-Aldrich) for the double fluorescence protocol. In total, three experimental rounds were conducted.

### Immunocytochemistry and image acquisition

Cells were stained by double fluorescence protocol as described by Ojala et al. (2012). Cells were fixed with 4% paraformaldehyde (PFA, Sigma-Aldrich) for 20 min at room temperature. Primary antibodies of cardiac Troponin T (TNT) (goat IgG, 1:2000, Abcam), α-actinin (AACT) (mouse IgG, 1:1500, Sigma-Aldrich) and myosin binding protein C (MYBP) (mouse IgG, 1:400, Santa Cruz) were used. Alexa Fluor 488 (anti mouse, 1:800, Thermo Fisher Scientific) and Alexa Fluor 568 (anti goat, 1:800, Thermo Fisher Scientific) were used as secondary antibodies. Prolong anti-fade gold reagent (1:2000, 20 min at RT, Thermo Fisher Scientific), containing 4′,6-diamidino-2-phenylindole (DAPI), was used to stain nuclei. Images were acquired through the PDMS membrane with Olympus IX51 inverted microscope equipped with 20x and 40x fluorescence air objectives and Olympus DP308 W camera (Olympus Corporation).

### Sarcomere orientation

For the orientation analysis of sarcomere structures in cardiomyocytes, an in-house developed software CytoSpectre (Kartasalo et al. 2015) was utilized. Orientation and detailed wavelength analytics with no segmentation was conducted to all immunofluorescence images. Circular statistical analytics were performed using the CircStat toolbox (Berens 2009) for MATLAB. Circular variance (CV) was used as an indicator for sarcomere orientation uniformity and Rayleigh test to confirm its statistical significance.

### Video acquisition and analysis

The proposed uniaxial stretching device enables live cell imaging and video recordings. In this study, we provide an example of capabilities of the stretching device for high-speed video recording and beating analysis. High-speed (60FPS), 1608 × 1208 resolution, videos of beating cardiomyocytes on PDMS devices were acquired in room temperature with Nikon (Tokyo, Japan) Eclipse TS100 inverted phase contrast microscope with 20× air objective and Imperx IGV-B1620 M camera. JAI software (Valby, DK) was used to control the camera and record the videos. Video analysis was performed using an in-house Cellvisus software developed by Ahola et al. (2014). Briefly, the software analyzes the beating motion of single cardiomyocytes or cell clusters providing a robust, noninvasive and label-free method to analyze mechanobiological functionality of cardiomyocytes derived from hiPSCs. The software is based on digital image correlation, minimum quadratic difference, and it calculates the times for each phase of beating to occur: contraction, relaxation and phases in between these two. It also calculates the beating frequency as beats per minute (BPM).

### Calcium imaging

Calcium imaging was used to demonstrate the capabilities of the uniaxial stretching device in cell functionality studies. To test calcium kinetics of uniaxially stretched cardiomyocytes on PDMS devices, cells were loaded with 4 μM Fura-2 AM (Molecular Probes, Life Technologies Ltd) for 30 min and de-esterified for 10 min in extracellular solution in 37 °C: (in mM) 137 NaCl, 5 KCl, 0.44 KH_2_PO_4_, 20 HEPES, 4.2 NaHCO_3_, 5 d-glucose, 2 CaCl_2_, 1.2 MgCl_2_ and 1 Na-pyruvate dissolved in MilliQ™ water. The pH of the extracellular solution was adjusted to 7.4 with NaOH. The Ca^2+^ kinetics of spontaneously beating CMs were imaged in room temperature with an inverted Nikon IX70 microscope using UApo/340 x20 air objective (Olympus Corporation) and recorded with ANDOR iXon 885 CCD camera (Andor Technology, Belfast, Northern Ireland) using 2 × 2 binning and synchronized with a Polychrome V light source (TILL Photonics, Munich, Germany). LiveAcquisition software (TILL Photonics) was utilized to control the light source and the camera during recording. Fura-2 AM was excited at 340 nm and 380 nm light, and the emission was recorded at 505 nm. For calcium imaging analysis, beating single cells or small clusters were selected as regions of interests (ROI), and background noise was subtracted before further processing with the LiveAcquisition software.

## Results

### Computational analysis and experimental characterization of the stretching device

**Fig. 2:**
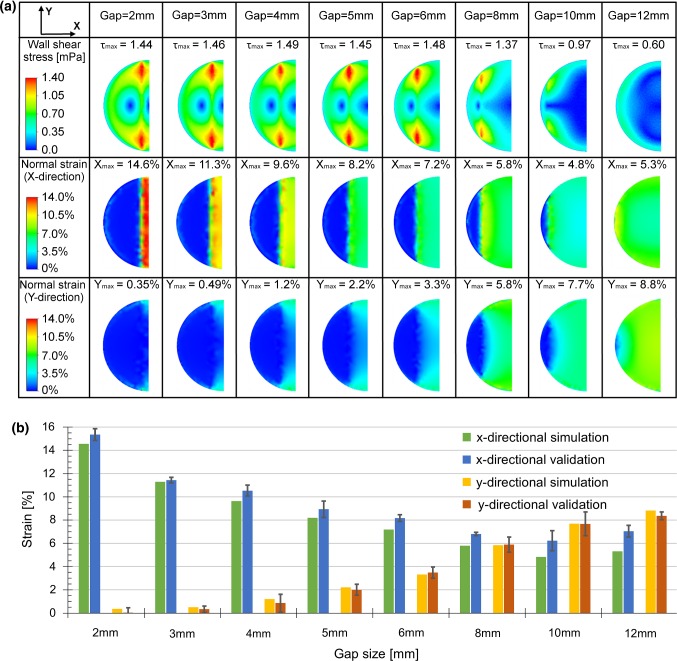
**a** Computational analysis illustrates the maximum wall shear stress and in-plane strain. **b***x*-directional and *y*-directional strain components for the varying gap widths. Simulated and measured strains components show a good correspondence and provide a validation for the numerical model. Based on these results, system with 5-mm gas width was selected for further cell experiments

Computational analysis was applied to quantify the wall shear stress and the in-plane strain field from the stretching devices with various gap widths varying from 2 mm to 12 mm. The results are shown in Fig. 2a. Computational analysis reveals that the wall shear stress is very small all over the well during the stretching. Figure 2a illustrates the peak wall shear stress that occurs during the cyclic sinusoidal stretching period. The peak wall shear stress is smaller than 1.5 mPa for all the cases. The shear stress analysis also reveals that by using large gap widths, the maximum shear stress is decreased (0.6 mPa).

In order to find a device design providing a large stretched cultivation area with high strain, the in-plane strain components of the membrane were evaluated with computational analysis and experimental characterization. In computational analysis, the in-plane strain was determined as the maximum value in the middle of the stretching area (1 mm × 1 mm area). Directional strain components (*x*- and *y*-axis) were defined to characterize the unidirectionality of the membrane. The *x*-direction describes the stretching direction, and the *y*-direction is undesired perpendicular deformation. The computational analysis was compared to experimental characterization of the stretching devices. The in-plane strain values and corresponding standard deviations for the varying gap widths are presented in Fig. 2b. The graph shows a good correspondence between computational simulations and experimental characterization providing a validation for the numerical model. It is clearly seen that the *x*-directional strain decreases and the *y*-directional strain increases when the gap width increases. Logically, by increasing the gap width between the two circular segments in a circular structure the percentage of the freely deforming area increases. Therefore, the device leans toward a more equiaxial strain when increasing the distance between the rigid layers and thus increasing the deforming area of the membrane. In order to achieve a sufficient uniaxility, the ratio between the *x*-directional and the *y*-directional strains should be larger than 4. At the same time, the cell area should be sufficiently large to provide enough cells for biochemical analysis. Therefore, the gap width of 5 mm was selected as it provides the desired functionality at an acceptable active cultivation area. All cell experiments were conducted with the stretching devices including 5 mm gap width.

### Cell morphology and orientation analysis

**Fig. 3:**
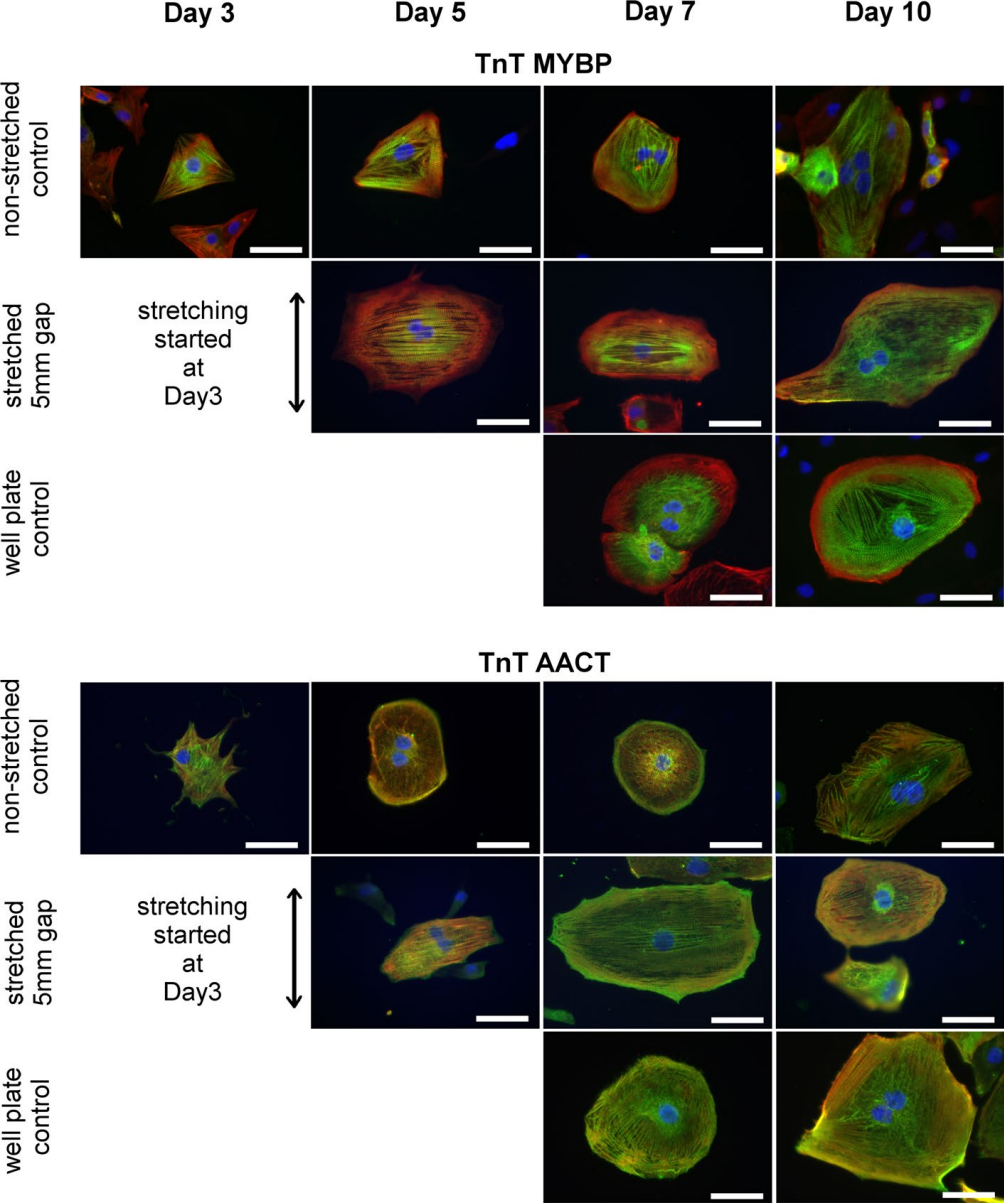
Sarcomere orientation under cyclic mechanical loading in stretched samples compared to static samples on non-stretched PDMS and 4-well plate controls at different time points. Day 3 images were taken the same day stretching was started. Axis of stretch in images is in vertical direction. Scale bar = 50 µm. TnT = red, MYBP = green, AACT = green, DAPI = blue

Cells were stained with anti-MYBP and anti-AACT antibodies to study cell morphology and sarcomere structure. Both are structural proteins of sarcomeres in cardiomyocytes, and they serve as cardiac markers. Figure 3 shows a morphology comparison between static and stretched cells at different time points. Cell morphology indicates successful attachment and survival of cells under the cyclic strain of 8% peak-to-peak value with 0.8 Hz. Also, more uniform sarcomere orientation, perpendicular to the applied strain, can be observed, which indicates improved structural maturation of cardiomyocytes.

**Fig. 4:**
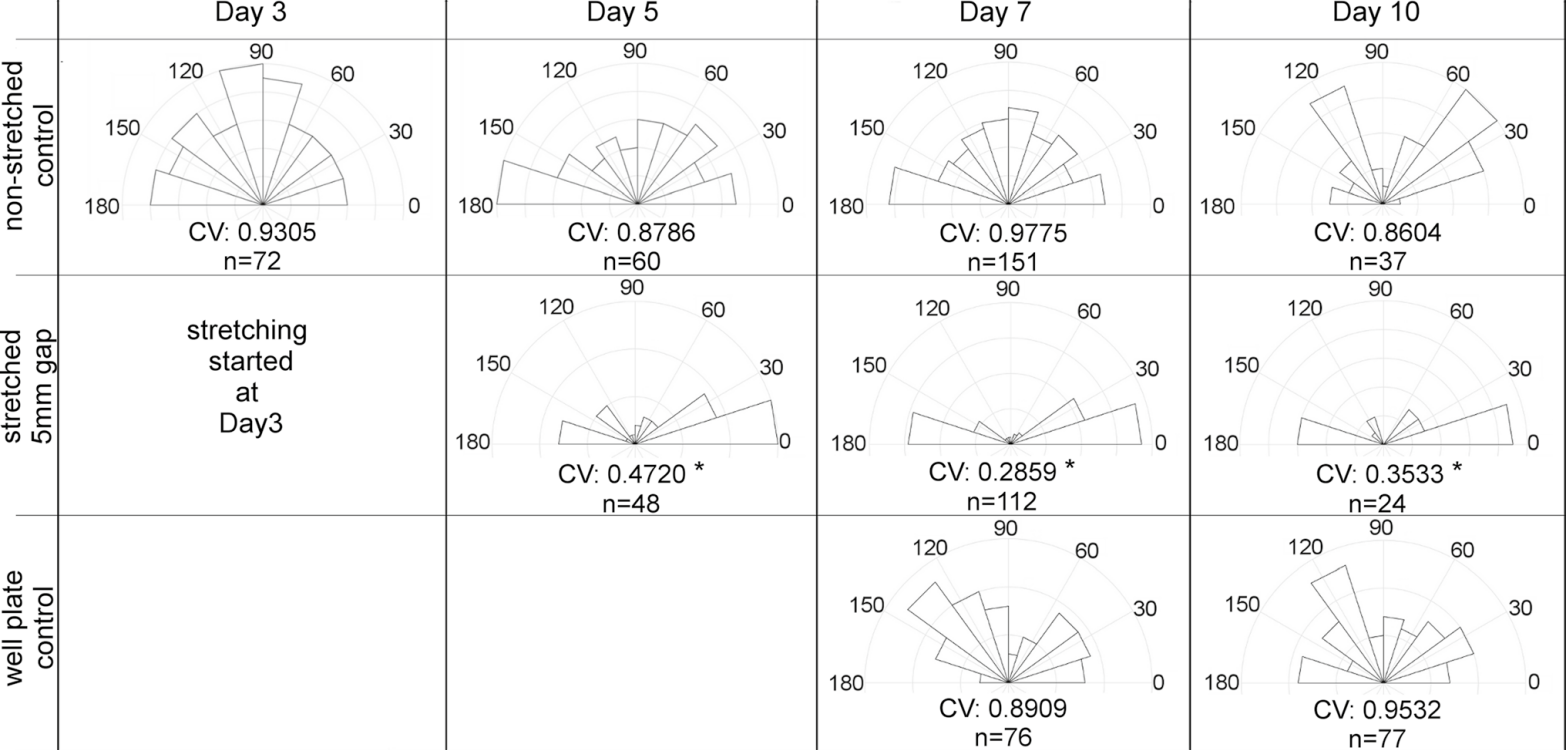
Orientation of sarcomere structures in non-stretched PDMS controls, unidirectional stretching devices, and 4-well plate controls. CV = circular variance where 0 is absolutely uniform and 1 is absolutely random orientation. *n* = number of cells analyzed for the study. Sector size indicates the proportion of cells oriented at that direction.*Rayleigh test shows statistical significance *p* < 0.001

Orientation analysis was performed using the CytoSpectre software to quantify the effect of unidirectional stretching on hiPSC-CMs. Number (*n*) of cells were analyzed in each time point and used for orientation analysis as shown in Fig. 4. Orientation analysis with circular variance clearly demonstrates the significant unidirectionality of sarcomere structures already after 2 days of stretching (Day 5). Analysis shows the statistically significant (*p* < 0.001) difference between stretched and non-stretch cells. Non-stretched control cells on PDMS and polystyrene surfaces do not show significant differences in sarcomere orientation and remain randomly oriented during the entire experiment. In addition, both stretched and non-stretched cultures show a healthy development of the cells.

### Imaging capabilities

**Fig. 5:**
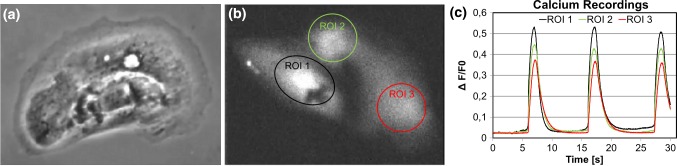
**a** Phase contrast image of a cardiomyocyte grown on PDMS stretching device illustrates the possibility of combining good-quality video imaging with further live cell analysis on the system. **b** Fluorescent image of cardiomyocytes loaded with Fura-2 calcium indicator illustrates the possibility of combining calcium imaging with the stretching device. **c** Calcium transients from a spontaneously beating cardiomyocyte on the stretching device. Background fluorescence is negligible

One important benefit of the proposed uniaxial stretching device is high-quality live cell imaging through the thin stretchable PDMS membrane. We performed experiments to demonstrate the capability of the proposed stretching device in two different image-based cell analysis methods: Phase contrast video-based beating analysis and fluorescent live cell calcium imaging. Both demonstrations were performed with spontaneously beating hiPSC-derived cardiomyocytes cultured in the uniaxial stretching devices with 5-mm gap. Figure 5a illustrates an example of the phase contrast image through the PDMS membrane of the stretching device. The image quality through the PDMS membrane is optically good for analyzing different characteristics of beating cells. We analyzed the beating behavior of cardiomyocytes with the Cellvisus analysis software (Video S1‡) that provides information on the contraction and relaxation times and on the beating frequency. We also show an example of calcium imaging through the stretchable PDMS membrane (Fig. 5b, c, video S2‡). The thin membrane does not disturb the calcium imaging and the background fluorescence is negligible. Therefore, the system provides a platform for further analysis of CMs’ calcium signaling that is known to regulate many biological processes in CMs (Keung et al. 2014). Furthermore, by providing possibility to combine different imaging modes (e.g., calcium and phase contrast imaging) simultaneously, it is possible to perform more versatile studies of CMs, as demonstrated in our previous study (Ahola et al. 2018).

## Discussion

Adult CMs are morphologically and functionally different from the hiPSC-CMs in vitro (Denning et al. 2016; Sun and Nunes 2017). Human iPSC-CMs under in vitro conditions do not express all physiologically relevant features present in mature adult CMs in vivo. In mature adult CMs, the sarcomere structure is more organized, sarcomere units are longer, and cells express elongated and oriented morphologies. Moreover, various functions such as calcium transients and electrophysiological features are different in mature adult CMs.

Typically, maturation of hiPSC-CMs in vitro is enhanced by applying specific biochemical inducers (Yang et al. 2014; Keung et al. 2014). However, this seems to be insufficient for mimicking adult CMs. Therefore, other cues, such as mechanical cues, are needed to further improve hiPSC-CM maturation in a laboratory environment. Improved maturation of hiPSC-CMs is an essential step before these cells can be used in therapies. Many features of the cardiomyocytes such as morphology, beating rate and electrophysiological markers can be followed by visualizing the cells with a microscope. For that purpose, the experimental arrangement should support visual inspection during the experimentations.

In this paper, we demonstrated and validated the functionality and experimental capabilities of our novel design of the uniaxial stretching device. Generally, uniaxial stretching is known to orientate the cells (Naruse et al. 1998; Lee et al. 2007; Jungbauer et al. 2008; Shao et al. 2013; Wang et al. 2016; Kamble et al. 2018). Uniaxial stretching affects sarcomere orientation of cardiomyocytes which further improves the structural maturation of the cells. Similar to Wang et al. (2016), we also demonstrated that the cells orientate perpendicular to the strain axis in our devices (Figs. 3, 4) and thus validated the biological functionality of the stretching device for long-term stretching experiments. In our experiments, the orientation of the sarcomeres was already seen in the first time point, after 2 days of stretching (Day 5), as shown in Fig. 4. Sarcomeres were slightly more oriented in the last two time points (Day 7 and 10) as indicated by the decrease in circular variance from 0.47 (Day 5) to 0.29 (Day 7) and 0.35 (Day 10). According to this study, 4 days of stretching is sufficient to gain the highest degree of orientation. In contrast, control cultures on the non-stretched PDMS and on the 4-well plates showed random orientation throughout the experiment period. The circular variances in these samples were clearly higher (from 0.86 to 0.98) than in the stretched devices. Furthermore, we performed stretching continuously for 7 days and did not see any problems with cell adhesion. In this study, cell adhesion was enhanced utilizing the covalent surface functionalization with ascorbic acid (Leivo et al. 2017). Ascorbic acid cross-links the gelatin covalently to the membrane and therefore provides a stable base for the cells during long-term stretching.

We also demonstrated the live cell imaging capabilities with an inverted microscope through the thin stretchable PDMS membrane. The stretching device is suitable for different live cell microscopy imaging techniques since the thin PDMS membrane did not produce background fluorescence and did not reduce the optical properties during live cell phase contrast imaging. In this study, we showed the capability for video-based beating analysis and fluorescent calcium imaging of hiPSC-derived CMs (Video S1‡ and S2‡). The videos were analyzed utilizing the Cellvisus video analysis software (Ahola et al. 2014). The combination of the Cellvisus software with the proposed unidirectional stretching device provides a unique possibility to analyze the beating behavior and possible abnormalities in different beating phases. Another notable advantage of the proposed stretching device is the capability to perform fluorescence live cell imaging. In this demonstration, we showed that calcium signaling is clearly seen and the background fluorescence is negligible (Fig. 5c). To summarize, the studies clearly demonstrated that the PDMS stretching device is highly suitable for different modes of live cell imaging. Furthermore, the possibility to combine different imaging modes enables deeper and more versatile studies, for example, to study simultaneously ionic (calcium signaling) and biomechanical functions (motion study based on phase contrast imaging) and the differences in their dynamics, as demonstrated in our previous study (Ahola et al. 2018).

The PDMS, stretching devices were compared to a commercially available state-of-the-art apparatus, Flexcell^®^, and its uniaxial UniFlex^®^ culture plates. Fundamentally, these two methods are similar to each other considering the application of mechanical loading via vacuum pressure formation. The most relevant difference between the two methods is related to the loading of the membrane. In the UniFlex^®^ culture plates, the vacuum pressure is applied below the membrane. The UniFlex^®^ membrane is supported with a loading post below the membrane that requires lubricant between the post and the membrane. The loading post and lubricant affect the imaging quality. In the UniFlex^®^ culture plate, imaging of the cells is impossible during the stretching because of the loading posts. Furthermore, imaging the UniFlex^®^ plate with an inverted microscope requires careful cleaning of the lubricants from the bottom of the membrane.

Another relevant difference is the thickness and compositions of the culture membrane: On UniFlex^®^ plates, the silicone-based double membrane is relatively thick (~ 1 mm) compared to our stretching PDMS device’s thin PDMS membrane (~ 0.12 mm). The thick UniFlex^®^ membrane absorbs and scatters light making detection difficult or even impossible with inverted microscopes, which are commonly used in cell culture laboratories. Moreover, we observed significant background fluorescence with the UniFlex^®^ plates during fluorescent imaging with DAPI (blue) and AlexaFluor 488 (green) stainings compared to the PDMS membrane, in which background fluorescence was nonexistent. Background fluorescence in UniFlex^®^ might come partly from residues of lubricant and partly from the thick membrane composition. For that reason, the UniFlex^®^ plate requires an upright microscope with water immersion objective for calcium imaging, whereas the PDMS device can be imaged also with inverted microscopes because of the thin, non-fluorescent PDMS membrane. Moreover, since the PDMS membrane is thin, this does not create any problems while mounting the sample on a microscope glass, compared to the UniFlex^®^ membrane, which requires 3D gel-like glue to hold a coverslip on top of the sample. This is especially important considering that oil immersion objectives for high magnification imaging require a short working distance. In addition, the PDMS device can be used as a platform for performing immunocytochemistry in small volumes. With UniFlex^®^ plates (6-well plate format), the sample area is rather large compared to the PDMS devices. This leads to larger consumption of reagents and resources (e.g., expensive antibodies for immunostaining). As a summary, compared to UniFlex^®^ culture plates the PDMS stretching devices provide more versatile platform for combined studies of stretching and imaging.

For the technical performance validation, we performed computational analysis of strain and wall shear stress on our stretching device. The analysis revealed that the medium moving in the culture well during the membrane stretching is negligible that lead to very small wall shear stress. The maximum simulated peak wall shear stress inside the entire culture well is smaller than 1.5 mPa for all different devices with different gap widths. This shear stress is substantially lower than reported for the commercial Flexcell^®^ device by Thompson et al. (2010). Thompson et al. simulated the shear stress inside the Flexcell^®^ BioFlex^®^ device and found the peak shear stress of 88 and 132 mPa for loading on post membrane strains of 3.8% to 6.7%, respectively, with 1 Hz stimulation frequency. Such a high shear stress in the BioFlex^®^ well exists due to the large reciprocating up-and-down movement of the vacuum-operated membrane in the cell culture side. The reciprocating membrane generates fluid movements inside the cell culture well which in turn creates relatively high shear stress. In the proposed stretching device, the reciprocating membrane locates outside the cell culture well and therefore the shear stress is substantially smaller. Such a high shear stress can promote the cell growth, proliferation or differentiation (Glossop and Cartmell 2009; Kreke et al. 2005). Also, already very low shear stress (10 mPa) can accelerate capillar*y*-like tube formation of endothelial progenitor cells as reported by Yamamoto et al. (2003). Therefore, using Flexcell^®^ culture plates the cells are actually exposed to two different mechanical stimulations: shear stress on top of the cells and substrate strain below the cells. In our device, the shear stress is negligible, and thus, the substrate strain is the only mechanical cue to influence cells during the stretching.

The mechanical strain of UniFlex^®^ membrane is limited because of the thick membrane attached to the well. Based on the UniFlex^®^ data sheet, the maximum achievable membrane strain is 7.9% with 90 kPa vacuum pressure. In our stretching device, the 8% unidirectional strain is achieved with 40 kPa using the 5 mm gap width. With a smaller gap width, the same unidirectional strain is achieved with lower vacuum pressure (8% strain; ~ 20 kPa; 2 mm gap width). Therefore, the intended strain is easier to achieve in our device. In conclusion, both systems have advantages and disadvantages, but our stretching devices exhibit more favorable imaging properties and provide mechanobiological stimulation solely from mechanical stretching of the cell substrate avoiding cues from shear stress.

## Conclusions

In this study, we validated and demonstrated the performance of unidirectional stretching device with hiPSC-derived CMs. The device provides pure mechanical strain without other mechanical cues for the cells. It also provides good optical properties for various imaging methods. We applied 8% cyclic sinusoidal strain to hiPSC-derived CMs and found them to increase their sarcomere orientation perpendicular to the axis of strain. Increased sarcomere orientation leads to structural maturation, which is an important feature considering the use of these cells in the future therapies. We also successfully demonstrated phase contrast and fluorescent live cell imaging with the PDMS devices without any background interference. In conclusion, the proposed PDMS stretching devices can be used for applying uniaxial strain in vitro and are applicable for demanding imaging applications. Furthermore, the flexible design of the system allows to run equiaxial and uniaxial strain experiments on hiPSC or other cells with a minimalistic change in fabrication protocol.

## Electronic supplementary material

Below is the link to the electronic supplementary material.

Supplementary material 1 (MP4 2839 kb)

Supplementary material 2 (MP4 11086 kb)
